# Genetic background dominates the susceptibility to ventricular arrhythmias in a murine model of β-adrenergic stimulation

**DOI:** 10.1038/s41598-018-20792-5

**Published:** 2018-02-02

**Authors:** Marisa Jelinek, Charlotte Wallach, Heimo Ehmke, Alexander Peter Schwoerer

**Affiliations:** 1 Department of Cellular and Integrative Physiology, University Medical Center Hamburg-Eppendorf, Hamburg, Germany; 2 DZHK (German Center for Cardiovascular Research), Partner site Hamburg/Kiel/Lübeck, Hamburg, Germany

## Abstract

In cardiovascular research, several mouse strains with differing genetic backgrounds are used to investigate mechanisms leading to and sustaining ventricular arrhythmias. The genetic background has been shown to affect the studied phenotype in other research fields. Surprisingly little is known about potential strain-specific susceptibilities towards ventricular arrhythmias *in vivo*. Here, we hypothesized that inter-strain differences reported in the responsiveness of the β-adrenergic pathway, which is relevant for the development of arrhythmias, translate into a strain-specific vulnerability. To test this hypothesis, we characterized responses to β-adrenergic blockade (metoprolol) and β-adrenergic stimulation (isoproterenol) in 4 mouse strains commonly employed in cardiovascular research (Balb/c, BS, C57Bl/6 and FVB) using telemetric ECG recordings. We report pronounced differences in the electrical vulnerability following isoproterenol: Balb/c mice developed the highest number and the most complex arrhythmias while BS mice were protected. Balb/c mice, therefore, seem to be the background of choice for experiments requiring the occurrence of arrhythmias while BS mice may give insight into electrical stability. Arrhythmias did not correlate with the basal β-adrenergic tone, with the response to β-adrenergic stimulation or with the absolute heart rates during β-adrenergic stimulation. Thus, genetic factors dominate the susceptibility to ventricular arrhythmias in this model of β-adrenergic stimulation.

## Introduction

In cardiovascular research, rodent models are commonly used to advance the understanding of mechanisms leading to and sustaining ventricular arrhythmias. Today, numerous mouse strains with differing genetic backgrounds are commercially available. Multiple studies have shown that the choice of the genetic background determines the studied phenotype, inter alia, in renal^1,2^, vascular^3,4^ and cardiovascular research^5–7^. Despite the high level of interest surprisingly little is known about the effects of the genetic background on the susceptibility to ventricular arrhythmias.

In a comprehensive study with 23 inbred mouse strains, Berthonneche *et al*. have reported that the β-adrenergic responsiveness to pharmacological interventions greatly varies between mouse strains^8^. The authors described a strong response in heart rate (HR) following i.p. injection of isoproterenol in Balb/c mice, while other strains such as FVB mice scarcely reacted to the same intervention. A high response to β-adrenergic activation has been linked to an increased risk for ventricular arrhythmias due to augmented Ca^2+^ loading of the sarcoplasmic reticulum and excessive activation of the Na^+^-Ca^2+^ exchanger^9–12^. This suggests that inter-strain differences in β-adrenergic responsiveness may translate into substantial differences in the susceptibility to ventricular arrhythmias between different mouse strains.

Accordingly, we hypothesized that mouse strains with a high response to a β-adrenergic activation are more vulnerable to ventricular arrhythmias than those with a low β-adrenergic responsiveness. To test this hypothesis, we selected 4 mouse strains which are commonly used in cardiovascular research and which are reported to differ substantially in their responses to β-adrenergic blockade and activation^7,8,13^: Balb/c and FVB mice with contrasting adrenergic responsiveness, as well as Black Swiss (BS) and C57Bl/6 (C57Bl/6) mice with intermediate phenotypes. Using telemetric ECG monitoring, we analyzed basal HR regulation and β-adrenergic responsiveness to blockade and stimulation *in vivo*. Susceptibility to ventricular arrhythmias was assessed following acute administration of the β-adrenergic agonist isoproterenol.

We report differences in the susceptibility to ventricular arrhythmias which are attributable to the genetic background. Specifically, Balb/c mice were most vulnerable to the intervention, while BS mice seemed to be protected against ventricular arrhythmias. Contrasting our hypothesis, the electrical stability did not correlate with the β-adrenergic responsiveness of the animals.

## Results

### Cardiac dimensions and function

**Figure 1:**
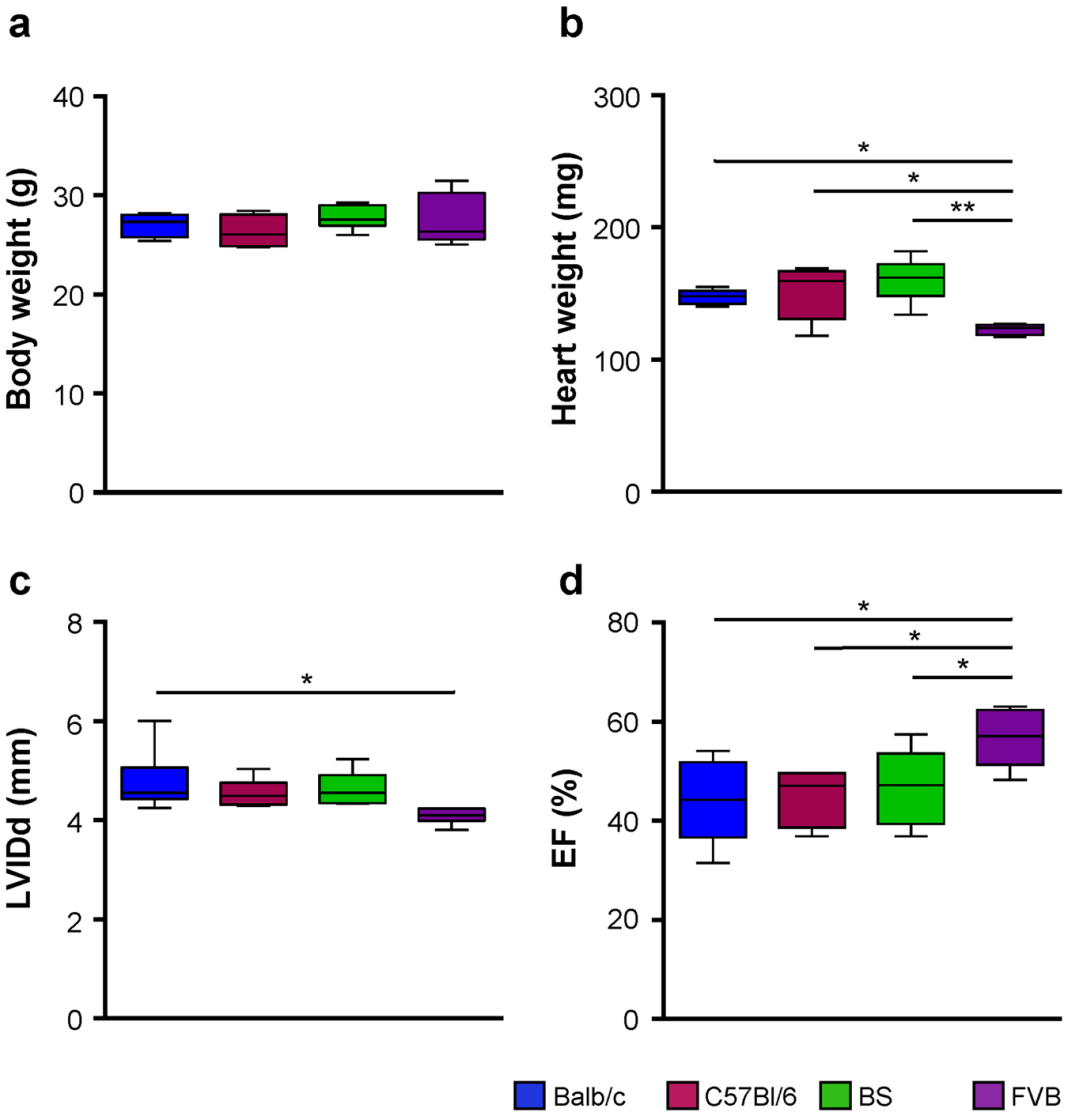
Cardiac geometry and function. (**a**) Body weight and (**b**) heart weight at the time of organ harvesting. (**c**) Diastolic left ventricular inner diameter (LVIDd) and (**d**) ejection fraction (EF). Data are given as median and 5–95 percentile. Balb/c n = 6, C57Bl/6 n = 6, BS n = 6, FVB n = 5. *p < 0.05; **p < 0.01; ***p < 0.001 (multiple comparisons were calculated by one-way ANOVA followed by Newman-Keuls post-hoc test).

To investigate cardiac dimensions and function of the 4 genetic backgrounds, all animals underwent echocardiography and were weighted regularly. Body weight and heart weight were recorded concurrently on the day of sacrifice. All animals of the 4 groups showed comparable body weight on this day, but heart weights were different, with significantly lighter hearts in the FVB group (Fig. 1a+b). Consistently, cardiac dimensions (e.g. LVID, Fig. 1c) and cardiac function (e.g. EF, Fig. 1d) were found to be similar in BS, C57Bl/6 and Balb/c mice. Hearts of FVB mice were smaller and displayed a higher EF (Fig. 1c+d).

**Figure 2:**
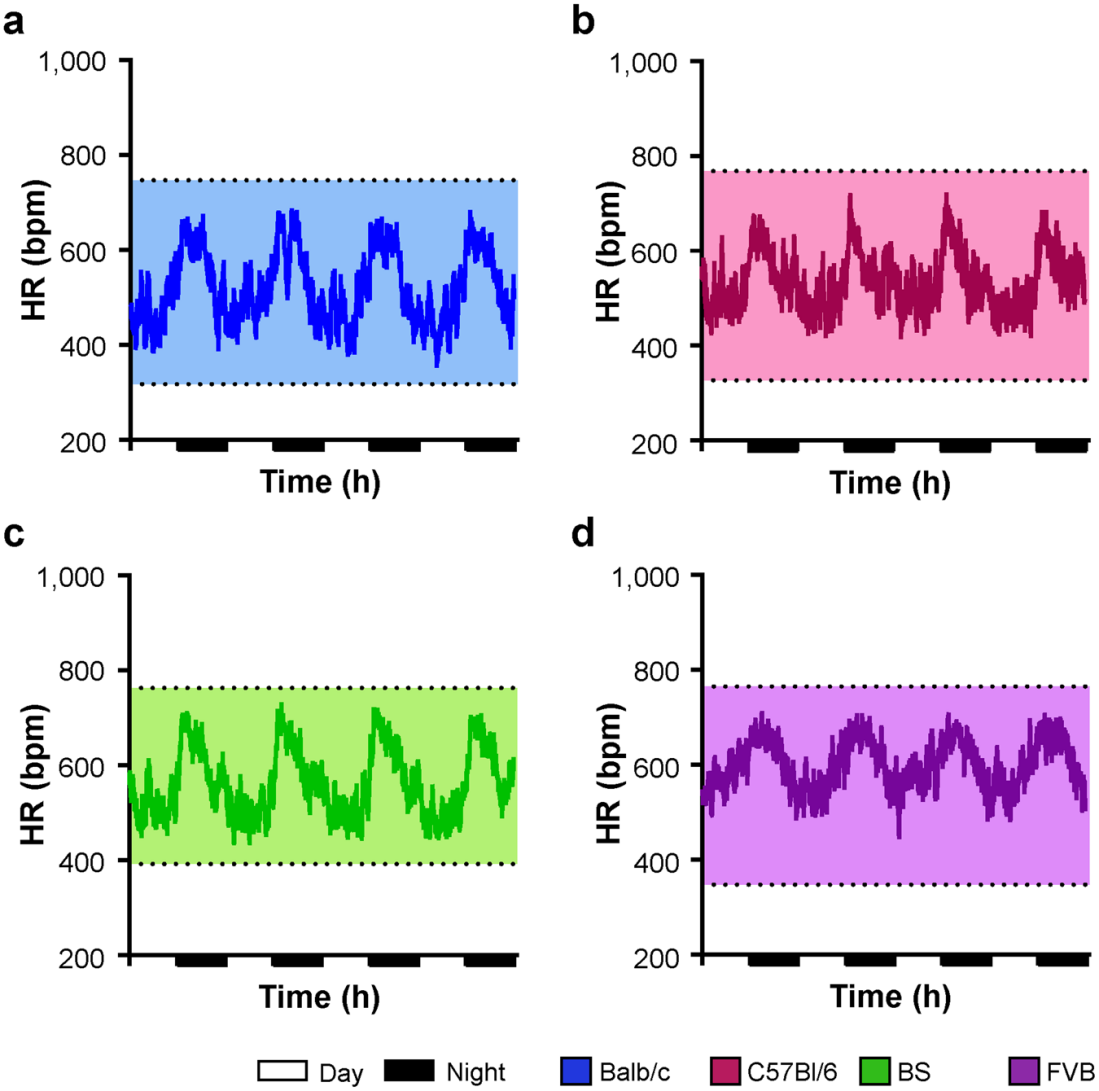
Heart rate regulation under baseline conditions. HR over 96 hours in (**a**) Balb/c, (**b**) C57Bl/6 mice, (**c**) BS mice and (**d**) FVB mice. For means of clarity, only mean values of each group are depicted. Upper and lower lines denote maximal and minimal HRs of each group. Day: 7AM-7PM, Night: 7PM-7AM. Balb/c n = 6, C57Bl/6 n = 6, BS n = 6, FVB n = 5.

**Figure 3:**
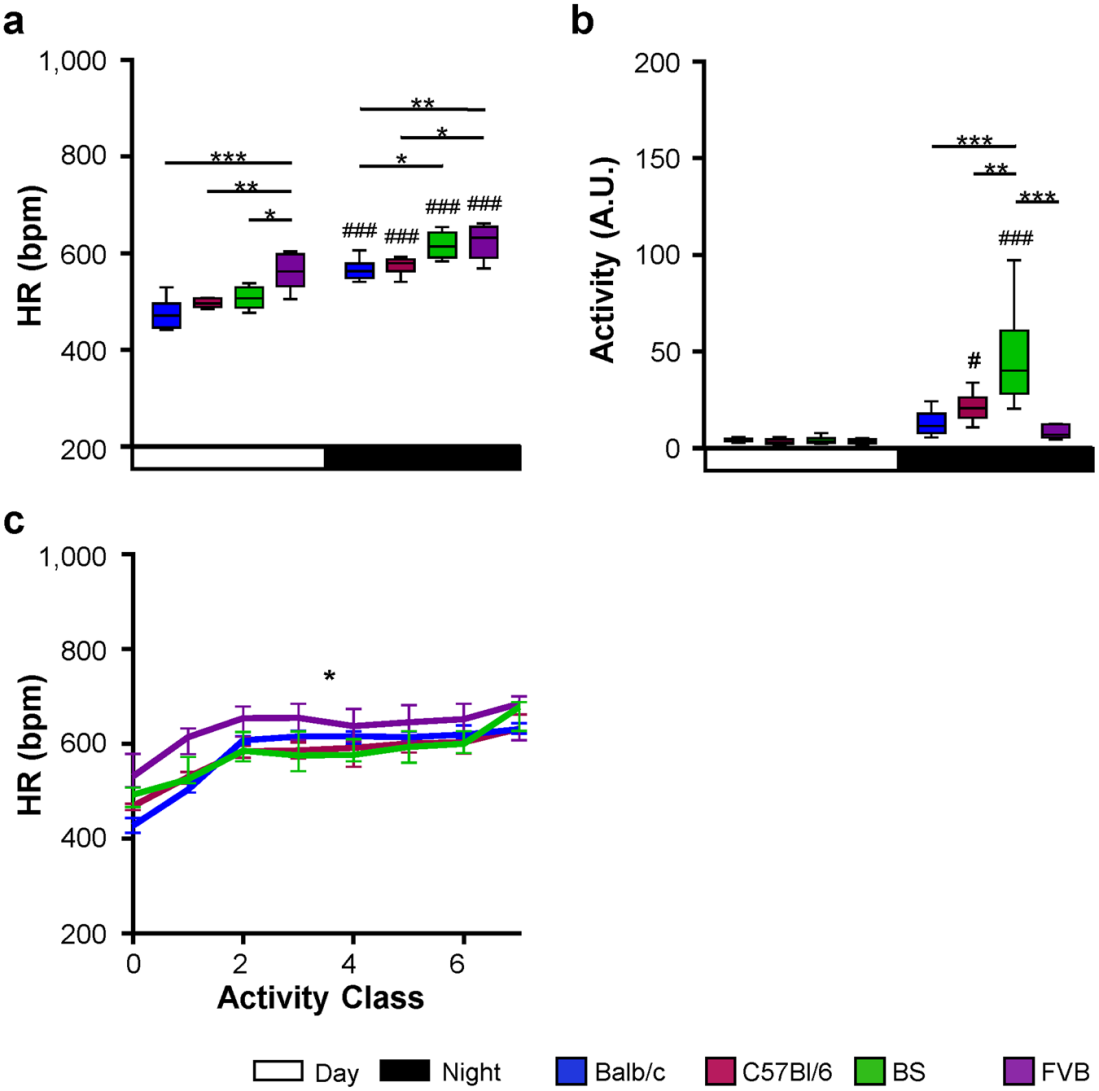
Heart rate and physical activity under baseline conditions. (**a**) HR and (**b**) physical activity during day- and night-time calculated from 96 hours ECG recordings as shown in Fig. 2. Activity was derived as a parameter (activity units; A.U.) from the telemetric transponders and corresponds to the physical activity of the animals in the cage. (**c**) HR normalized to activity. For this, the absolute activity units (A.U.) were binned in the following classes: 0 = 0 A.U., 1 = 0–5 A.U., 2 = 5–10 A.U., 3 = 10–15 A.U., 4 = 15–20 A.U., 5 = 20–25 A.U., 6 = 25–30 A.U., 7 = 30–300 A.U. HR values were then averaged according to the corresponding activity. Day: 7AM-7PM, Night: 7PM-7AM. Data is given as median and 5–95 percentile (**a**,**b**) and as median and interquartile range (**c**). Balb/c n = 6, C57Bl/6 n = 6, BS n = 6, FVB n = 5. *p < 0.05; **p < 0.01; ***p < 0.001 vs. another strain; #p < 0.05 vs. day, ###p < 0.001 vs. day (multiple comparisons were calculated by two-way ANOVA followed by Sidak post-hoc test).

Investigation of HR was performed using telemetric ECG transmitter. ECGs of untreated mice were recorded over 96 hours. All genotypes displayed stable day-night rhythm with higher HRs during the night (Fig. 2a–d). The highest naturally occurring HRs were in the range of 750–780 bpm and were comparable among the groups (Table 1). On the other hand, Balb/c mice had the lowest occurring HRs during the observation period (Table 1). When calculated over 96 hours, FVB and BS mice showed the highest mean HRs during day and night (Fig. 3a).

**Table 1:** Heart rate under baseline conditions and under β-adrenergic blockade.

	Balb/c	C57Bl/6	BS	FVB
*Baseline*				
minimal HR	317 ± 9 bpm	327 ± 27 bpm	392 ± 8 bpm	372 ± 23 bpm
			p < 0.05 vs. Balb/c	
maximal HR	747 ± 7 bpm	768 ± 7 bpm	763 ± 8 bpm	774 ± 17 bpm
*Metoprolol*				
minimal HR	288 ± 7 bpm	278 ± 34 bpm	342 ± 17 bpm	376 ± 11 bpm
				p < 0.05 vs. Balb/c p < 0.05 vs. C57Bl/6
maximal HR	706 ± 6 bpm	719 ± 5 bpm	727 ± 7 bpm	765 ± 17 bpm
				p < 0.01 vs. Balb/c p < 0.05 vs. C57Bl/6 p < 0.05 vs. BS

Minimal and maximal naturally occurring HRs per group under baseline conditions and under chronic administration of metoprolol. Values were calculated from 96 hours of ECG recordings. Data is given as mean ± SEM. Multiple comparisons were calculated using one-way ANOVA followed by Newman-Keuls post-hoc test. Balb/c n = 6, C57Bl/6 n = 6, BS n = 6, FVB n = 5.

Since HR depends on the physical activity of the animals, the inter-strain differences in HR could be caused by differing activity levels (Fig. 3b). During the activity phase, BS mice were significantly more active than all other strains (Fig. 3b). The higher observed HRs in BS than in C57Bl/6 and Balb/c mice are, therefore, paralleled by a higher activity level in these mice (Fig. 3a+b). In contrast, FVB mice, which displayed similarly high HRs as BS mice during day and night-time were less active. Accordingly, when normalized to the activity, FVB mice had significantly higher HRs over all levels of activity than the other strains (Fig. 3c). Thus, overall high HRs in FVB mice cannot be explained by a higher physical activity.

### β-adrenergic modulation

As strain-dependent HRs could be affected by differences in the sympatho-vagal balance, the basic β-adrenergic response was investigated in the 4 mouse strains. Accordingly, we treated the animals successively with both, a β-adrenergic blocker (metoprolol, 100 mg/kg body weight per day, p.o.) and a β-adrenergic stimulator (isoproterenol, 2 mg/kg body weight, i.p.).

**Figure 4:**
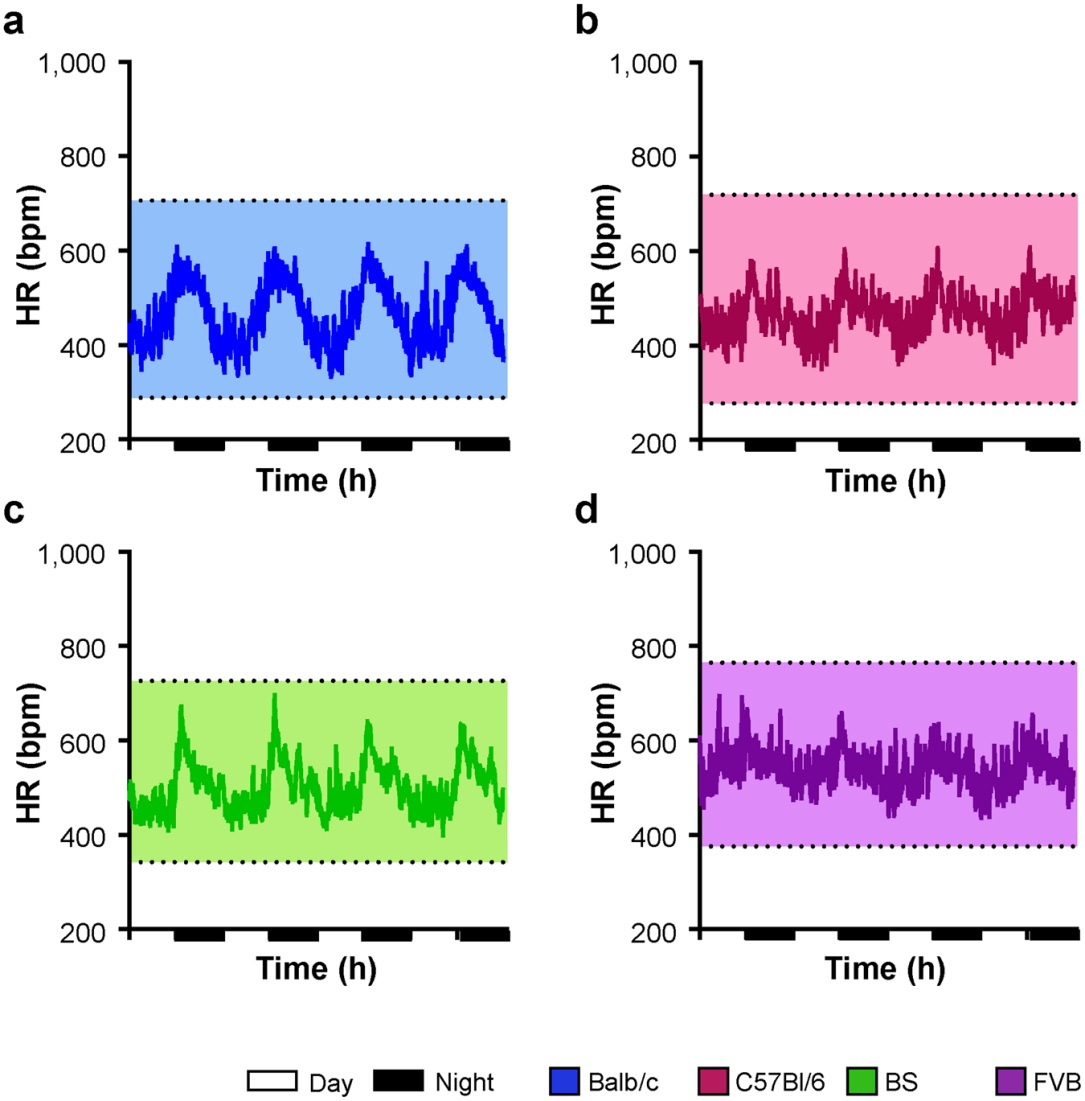
Heart rate regulation under β-adrenergic blockade. HR under treatment with metroprolol over 96 hours in (**a**) Balb/c, (**b**) C57Bl/6 mice, (**c**) BS mice and (**d**) FVB mice. For means of clarity, only mean values of each group are depicted. Upper and lower lines denote maximal and minimal HRs of each group. Day: 7AM-7PM, Night: 7PM-7AM. Balb/c n = 6, C57Bl/6 n = 6, BS n = 6, FVB n = 5.

**Figure 5:**
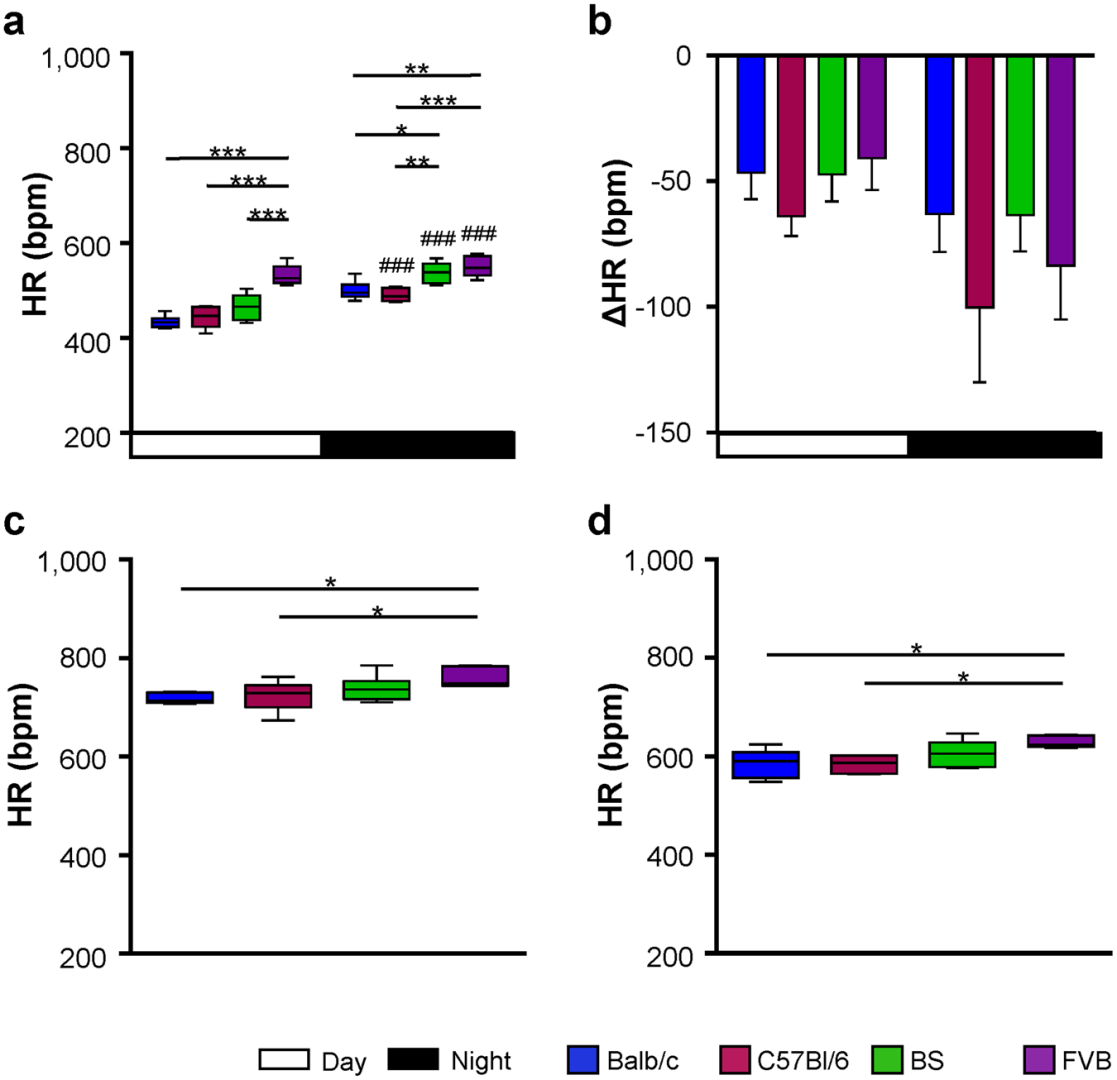
Heart rate under β-adrenergic modulation. (**a**) HRs under treatment with metoprolol during day- and night-time derived from 96 hours ECG recordings shown in Fig. 4. (**b**) HR reduction (ΔHR) calculated as the difference between mean HRs under baseline conditions and under β-adrenergic blockade. (**c**) Maximal HRs and (**d**) mean HRs over 5 hours following isoproterenol injection. Day: 7AM-7PM, Night: 7PM-7AM. Data is given as median and 5–95 percentile (**a**,**c**,**d**) and as median and interquartile range (**b**). Balb/c n = 6, C57Bl/6 n = 6, BS n = 6, FVB n = 5. *p < 0.05; **p < 0.01; ***p < 0.001 against another strain; ###p < 0.001 against day. Multiple comparisons were calculated by (**a**) two-way ANOVA followed by Sidak post-hoc test, and (**b**–**d**) one-way ANOVA followed by Newman-Keuls post-hoc test.

Under treatment with metoprolol, the HR was reduced in all strains (Fig. 4a–d). On average, FVB and BS mice had higher mean HRs than C57Bl/6 and Balb/c mice (Fig. 5a). The mean HR during day-time was ~50 bpm lower than under baseline conditions (Fig. 5b, day-time). As expected, the reduction in HR was more pronounced during the activity phase of the animals (Fig. 5b, night-time). Notably, metoprolol reduced the HRs comparably in all strains, indicating similar activation of β-adrenoceptors under basal conditions. The strain-dependent gradient in HRs observed under baseline conditions was not affected by the β-adrenergic blockade with FVB mice showing the highest and Balb/c and C57Bl/6 mice showing lower HRs (Fig. 5a, Table 1).

Following acute injection of isoproterenol, HR increased in all mice. The highest resulting HRs were observed in FVB mice (Fig. 5c). The mean HR following isoproterenol injection was also different between the groups, with a significantly higher HR in the FVB group compared to C57Bl/6 and Balb/c (Fig. 5d). Again, the overall strain-dependent gradient in HRs observed under baseline conditions and under β-adrenergic blockade was not affected by the β-adrenergic stimulation.

### Susceptibility to ventricular arrhythmias under β-adrenergic stimulation

To investigate potential inter-strain differences in the susceptibility to ventricular arrhythmias, ECGs were analyzed in a time frame of 5 hours after NaCl/isoproterenol injection. Following sham-injection of NaCl, only in single mice of all groups a very low number of premature ventricular beats could be detected (data not shown). Occurring arrhythmias in the presence of isoproterenol can, therefore, be fully attributed to the β-adrenergic stimulation.

**Figure 6:**
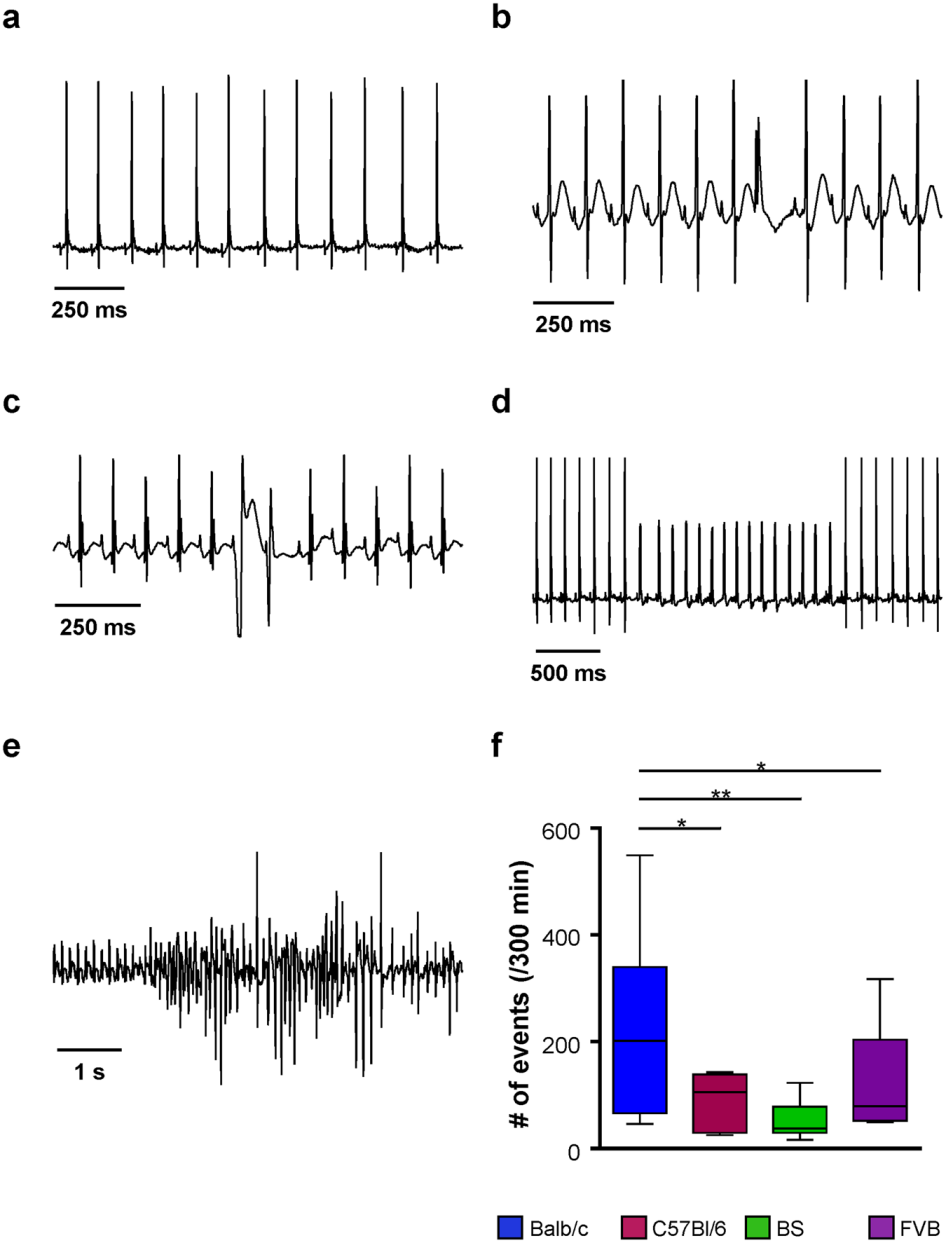
Representative ECGs and occurrence of ventricular arrhythmias under β-adrenergic stimulation. (**a**) Sinus rhythm, (**b**) premature ventricular beats (PVB), (**c**) coupled beats, (**d**) ventricular tachycardic events (VT) and (**e**) ventricular fibrillation events (VF). (**f**) Number of total arrhythmic events (sum of PVBs, coupled beats, VT and VF) over 5 hours following isoproterenol injection. Data are given as median and 5–95 percentile. Balb/c n = 6, C57Bl/6 n = 6, BS n = 6, FVB n = 5. *p < 0.05; **p < 0.01 (multiple comparisons were calculated by Kruskal-Wallis test followed by Dunn’s post-hoc test).

Under treatment with isoproterenol ventricular arrhythmias could be detected in all animals. Premature ventricular beats (PVB; Fig. 6b), coupled beats (e.g, couplets, triplets, Fig. 6c), ventricular tachycardic events (VT; Fig. 6d) and ventricular fibrillation (Fig. 6e) emerged. In total, Balb/c mice developed the highest burden of ventricular arrhythmias (Fig. 6f). Furthermore and in contrast to the other genotypes, all Balb/c mice developed all subtypes of ventricular arrhythmias and had significantly more tachycardic events than BS and C57Bl/6 mice (Table 2). Finally, Balb/c mice were the only group that developed ventricular fibrillation (Table 2). Taken together, we found a strain-specific gradient in electrical vulnerability (Table 2). In Balb/c mice, a ~4 fold higher arrhythmic burden was detected than in BS mice. FVB and C57Bl/6 mice ranked in between with intermediate arrhythmic phenotypes.

**Table 2:** Occurrence of ventricular arrhythmias under β-adrenergic stimulation.

	PVB		Coupled beats		VT		VF	
	Events	Animals	Events	Animals	Events	Animals	Events	Animals
Balb/c	158.2 ± 54.1	(6/6)	46.7 ± 18.0	(6/6)	14.0 ± 5.4	(6/6)	4.7 ± 1.7	(4/6)
					p < 0.05 vs. FVB p < 0.05 vs. C57Bl/6 p < 0.05 vs. BS		p < 0.01 vs. FVB p < 0.01 vs. C57Bl/6 p < 0.01 vs. BS	
FVB	89.8 ± 35.2	(5/5)	25.2 ± 14.3	(4/5)	4.0 ± 1.7	(4/5)	0.0 ± 0.0	(0/5)
C57Bl/6	72.8 ± 17.0	(6/6)	18.2 ± 5.5	(6/6)	1.2 ± 0.7	(3/6)	0.0 ± 0.0	(0/6)
BS	40.2 ± 10.4	(6/6)	11.2 ± 4.3	(5/6)	0.5 ± 0.3	(2/6)	0.0 ± 0.0	(0/6)

Total number of arrhythmic events during β-adrenergic stimulation and fraction of animals in which these arrhythmias occurred per group and subclass of arrhythmic event. Premature ventricular beats (PVB); coupled beats, e.g. couplets; ventricular tachycardic events (VT) and ventricular fibrillation (VF). Data is given as mean ± SEM. Statistical significances refer to the number of arrhythmias per group and were calculated using one-way ANOVA followed by Newman-Keuls post-hoc test. Balb/c n = 6, C57Bl/6 n = 6, BS n = 6, FVB n = 5.

## Discussion

In cardiovascular research, a plethora of mouse strains with differing genetic backgrounds are commercially available and used today. The genetic background has repeatedly been shown to markedly affect cardiac function under physiological as well as under pathophysiological conditions^5–7,14,15^. Using an *in vivo* murine model of β-adrenergic activation, we here report that the genetic background also determines the susceptibility to ventricular arrhythmias in healthy animals. Following stimulation, Balb/c mice were significantly more susceptible to arrhythmias than FVB, C57Bl/6 and BS mice. Moreover, in Balb/c mice more complex arrhythmias such as VT and VF developed in a larger fraction of the animals than in the other strains. Conversely, BS mice were found to be particularly resistant against ventricular arrhythmias with only few arrhythmic events and no episodes of VT or VF observed during the β-adrenergic challenge.

Previous studies addressing potential inter-strain differences in the propensity to ventricular arrhythmias have reported inconsistent data. In anesthetized animals, Maquire *et al*. were substantially more successful to induce VTs during electrical stimulation in FVB and BS mice than in C57Bl/6 mice, respectively^16^. Using *ex vivo* Langendorff hearts, Waldeyer *et al*. found a higher incidence of arrhythmias in C57Bl/6 mice than in FVB mice^17^. In the current study, an *in vivo* experimental approach was chosen to assess inter-strain differences in intact animals with an unaffected autonomic regulation. In this context, Balb/c mice were more prone for cardiac arrhythmias induced by isoproterenol than FVB, C57Bl/6 and BS mice. This should be considered when planning experiments which rely on the stable occurrence of ventricular arrhythmias, e.g. in transgenic animals. Moreover, in combination with BS mice which present a particularly high protection against ventricular arrhythmias, the relatively sensitive Balb/c mice would be of great value to further investigate the genetic basis underlying ventricular arrhythmias, e.g. by using a reciprocal backcross strategy^18^.

An excessive β-adrenergic stimulation increases the occurrence of severe ventricular arrhythmias^12,19^. Mechanistically, this could be mediated via the β-adrenergic modulation of the ventricular Ca^2+^ balance. The systolic Ca^2+^ transient of ventricular cardiomyocytes is strongly influenced by the Ca^2+^ content of the sarcoplasmic reticulum^9^. Under β-adrenergic stimulation, the sarcoplasmic Ca^2+^ load is augmented by a net increase in sarcoplasmic Ca^2+^ ATPase activity and a larger Ca^2+^ influx through L-type Ca^2+^ channels. This, in turn, causes a disproportional increase in the amplitude of the systolic Ca^2+^ transient and mediates the positive inotropy. As pointed out by Eisner and Trafford, under these conditions the Ca^2+^ efflux through the Na^+^-Ca^2+^ exchanger must also increase to keep the Ca^2+^ fluxes in balance^9–11^. Due to its stoichiometry, activation of the Na^+^-Ca^2+^ exchanger depolarizes the myocyte, thereby exerting a pro-arrhythmic effect^20^. Following this concept, we hypothesized that the occurrence of ventricular arrhythmias correlates with the inter-strain differences in β-adrenergic responsiveness reported by Berthonneche *et al*.^8^. In the study of Berthonneche *et al*. the effect of a β-adrenergic stimulation inversely correlated with the basal HRs. In Balb/c mice which revealed the lowest HRs under baseline conditions, the increase in HR following isoproterenol injection was several times higher than in FVB mice which had high control HRs. Compared to these strains, C57Bl/6 as well as BS mice displayed intermediate phenotypes. In the current study, using continuous ECG recordings under non-restraint conditions, the differences in basal HRs reported by Berthonneche *et al*. could be generally confirmed in all 4 strains. In the presence of isoproterenol, however, these differences persisted due to a similar and strain-independent increase in HR. In contrast to the study of Berthonneche *et al*. the response to an identical β-adrenergic stimulation was, therefore, identical in all strains. While this contrasting β-adrenergic responsiveness may be caused by different technical approaches to measure the HR, the reason for this mismatch remains unresolved. Under the assumption that the steady state production of cAMP in sinus node cells and in ventricular cardiac myocytes is comparably regulated by β-adrenergic stimulation, the similar increase in HR implicates that the cytosolic cAMP concentrations in ventricular myocytes is increased by a comparable degree in all strains. Thus, its downstream targets, e.g. the L-type Ca^2+^ channels and the sarcoplasmic Ca^2+^ ATPase, should be similarly activated leading to comparable increases in sarcoplasmic Ca^2+^ contents. Nonetheless, the number and severity of observed ventricular arrhythmias was clearly different in the strains. A variety of structural and functional differences between the strains, including cell-cell interaction, cell metabolism and ion channel expression, is conceivable which might contribute to the strain-specific susceptibility to arrhythmias. Notably, it could also be the consequence of inter-strain differences in cellular Ca^2+^ handling. Shah *et al*. reported that Balb/c mice displayed a higher level of Ca^2+^ sparks and Ca^2+^ leakage from the sarcoplasmic reticulum than C57Bl/6, FVB and SV129 mice^14^. A higher instability of the sarcoplasmic reticulum could well increase the risk for ventricular arrhythmias following isoproterenol injection and could, therefore, underlie the high arrhythmic burden of Balb/c mice observed in the current study. Shah *et al*. discussed strain-specific differences of PKA and CaMKII dependent ryanodine receptor phosphorylation or in myofilament sensitivity to Ca^2+^ as underlying mechanisms^14^. This concept is supported by more recent studies that addressed genetic variants underlying the phenotypic heterogeneity in the response to isoproterenol^21,22^. Using RNAseq analysis, Prunotto *et al*. reported that Ca^2+^ signaling and contractile fibers are among the most differently regulated gene clusters^22^. Similarly, Rau *et al*. performed a GWAS analysis in inbred mice from the hybrid mouse diversity panel and identified several genes with well-established roles in cardiac physiology including the key Ca^2+^ cycling regulator phospholamban^21^. The concept that strain-specific effects of isoproterenol may at least in part result from strain-specific differences in cellular Ca^2+^ handling appears highly attractive and should be addressed in further studies.

The susceptibility to ventricular arrhythmias could also be determined by the basal tone of β-adrenoceptors. Animals with an intrinsically low tone could develop less ventricular arrhythmias than mice with a high tone. This concept is supported by the observation that the arrhythmic burden increases with HR in heart failure patients^23,24^. In mice, HR is strongly affected by the sympathetic tone whereas the vagal influence mainly affects HR variability in the high frequency range^25^. The inter-strain differences in HRs observed under baseline conditions could indicate corresponding differences in the basal tone of β-adrenoceptors which could, therefore, underlie the differing electrical vulnerability. This was addressed by a chronic β-adrenergic blockade with metoprolol which gives a good estimate of the basal sympathetic tone^25^. We found that administration of metoprolol similarly reduced the HR irrespective of the circadian rhythm and, importantly, independent of the strain. This suggests that the basal sympathetic tone mediated by the β-adrenergic receptors does not differ between the 4 mouse strains investigated and cannot explain the observed inter-strain differences in the occurrence of arrhythmias. While these considerations argue against a pronounced strain-dependent heterogeneity in the β-adrenergic receptors, differences in the coupling and/or downregulation of β-adrenergic receptors which have been reported during long-term treatment with isoproterenol cannot be ruled out^26^. The relevance of these functional differences during acute isoproterenol injection, as performed in the current study, remains to be clarified.

The current study was designed to addresses the influence of the genetic background on the electrical vulnerability in healthy animals. Using isoproterenol injections, we could indeed demonstrate a variance in the electrical phenotype attributable to the genetic background. It is important to note that translation of the background specific effects into murine models of cardiac disease or into patients is not possible without further experiments. Under clinical conditions, a complex combination of several pathomechanisms underlies the development of ventricular arrhythmias. Furthermore, the phenotypes of experimental models of cardiovascular disease *per se* depend on the genetic background^1–7^. The background specific variance in the electrical phenotype can, therefore, be expected to be combined with the background specific variance in the phenotype of the cardiovascular disease. For example, the cellular remodeling process following coronary artery ligature has been shown to strongly depend on the mouse strain^7^. Since the development of ventricular arrhythmias strongly depends on the cardiac remodeling process, a β-adrenergic overstimulation following myocardial infarction may not result in a similar genotype dependency of electrical vulnerability as in healthy animals. Further experiments are, therefore, necessary to address this clinically highly relevant issue.

Ventricular arrhythmias were provoked by the standard protocol of acute injection of the β_1_- and β_2_-adrenoceptor agonist isoproterenol. To address potential effects of isoproterenol which are independent of cardiac β_1_- and β_2_-adrenoceptor and the individual role of these receptors in the context of different mouse strains, knock out mice lacking β_1_- and/or β_2_-adrenoceptors would be highly useful^27,28^.

In conclusion, we provide evidence that the occurrence of ventricular tachyarrhythmias following isoproterenol injection in 4 commonly used mouse strains is strain-specific. Balb/c mice developed the highest arrhythmic burden and the highest amount of potentially lethal VT and VF during the intervention while, on the other hand, BS mice were most protected against ventricular arrhythmias. Implications should be considered in experimental models addressing ventricular arrhythmias: Balb/c mice can be used with advantage in studies which rely on the stable occurrence of ventricular arrhythmias. Moreover, this relatively sensitive strain could also be valuable in combination with the electrically more stable BS mice to address the genetic basis for ventricular arrhythmias. Neither the β-adrenergic responsiveness nor the absolute heart rates during β-adrenergic stimulation nor the basal β-adrenergic tone determined the susceptibility to ventricular arrhythmias in the current study. Thus, genetic factors appear to dominate the susceptibility to ventricular arrhythmias in this murine model of β-adrenergic stimulation.

## Material and Methods

### Ethics statement

This study was carried out in strict accordance with the recommendations in the Guide for the Care and Use of Laboratory Animals of the National Institutes of Health (NIH; Publication No. 85-23, revised 1985). The protocol was approved by the local authorities (BGV, Freie und Hansestadt Hamburg; Germany, 103/11). All surgery was performed under deep sevoflurane anesthesia, and all efforts were made to minimize suffering.

### Study protocol

Mice of 4 widely used genetic strains were used: Balb/c, C57Bl/6, BS and FVB. All animals were bred in the animal facility of the Medical University Center Hamburg Eppendorf, strains originated from Charles River (Sulzfeld, Germany). In total, 23 male mice of a mean age of 9 (7–10) weeks were successfully implanted with telemetric ECG-transmitters and completed the study protocol (Balb/c n = 6, C57Bl/6 n = 6, BS n = 6, FVB n = 5). After a recovery time of 14 days, baseline ECGs were recorded over 96 hours, followed by echocardiography in each animal. β-adrenergic stimulation was conducted using a cross-over design model of isoproterenol injection with a control injection of NaCl. Following this procedure, animals were orally treated with the β-adrenergic antagonist metoprolol over a period of 2 weeks followed by a second ECG recording of 96 hours.

### Electrocardiographic recordings

ECGs were recorded in conscious, untethered mice using a telemetric system (Data Sciences International, St. Paul, MN, USA) as described previously^29,30^. Mice were anesthetized (sevoflurane, 3–5%) and a transponder was implanted subcutaneously (PhysioTel® TA11ETA-F10, Data Sciences International, St. Paul, MN, USA). The negative lead was placed in the area of the right shoulder and the positive lead left of the xiphoid space and caudal the rib cage approximating an Einthoven Lead II configuration. Analgesia and postoperative care followed institutional guidelines and included administration of carprofen (5 mg/kg body weight s.c., intraoperatively) and metamizol (300 mg/kg body weight, p.o., 48 h postoperatively). Animals were housed in individual cages on a receiver plate and allowed free access to food and water. Day-night rhythm was established with day-time (lights on) between 07:00AM and 07:00PM. Room temperature and humidity were controlled at 20–24 °C and 45–65%, respectively.

During long-term recordings, ECG waveforms were recorded at a sampling rate of 1 kHz using Dataquest A.R.T. (v 4.0, Data Sciences International) for 1 min every 5 mins. Physical activity was stored as a parameter (activity units, A.U.) every 5 mins. ECG waveforms during acute β-adrenergic stimulation were recorded continuously using iox2 (v.2.5.1.35, emka TECHNOLOGIES S.A., Paris, France). ECG recordings were analyzed using Dataquest A.R.T. and ecgAUTO (v.2.5.1.35, emka TECHNOLOGIES S.A., Paris, France).

### Treatment with β-adrenoceptor antagonist

On the basis of a metoprolol dose-response study (0–1000 mg/kg body weight per day), animals received the β-adrenoceptor blocking agent metoprolol (metoprolol-tartrate, SIGMA, M5391) orally in a dosage of 100 mg/kg body weight per day, solved in drinking water, over a period of 2 weeks consecutively.

### Treatment with β-adrenoceptor agonist

β-adrenergic stimulation was conducted using a cross-over design model of 2 consecutive injections of either the β-adrenoceptor agonist isoproterenol (isoproterenol, SIGMA, I5627; 2 mg/kg body weight, solved in 5 ml NaCl per kg body weight, i.p.) or NaCl (0.9%, 5 ml/kg body weight, i.p.) with an interval of 30 min between the injections^29^. Each mouse underwent both treatments at a random order with an interval of one week between the tests. Baseline ECG recordings were started 30 min before the first injection and ECGs were continuously recorded over an observation period of 300 min. In this time frame, the effects of the β-adrenoceptor agonist isoproterenol fully declined in all animals.

### Arrhythmia detection and classification

Ventricular arrhythmias were detected using a multiple approach concept with ecgAUTO (v.2.5.1.35, emka TECHNOLOGIES S.A., Paris, France). ECG waveforms were analyzed and ECG complexes were identified using animal-specific waveform libraries. Sections where waveform matching failed, e.g. due to predominant artefacts by high skeletal muscle activity, were categorized manually. Based on the ECG waveform (e.g. P waves, shape of the QRS complexes) abnormal beats were classified semi-automatically with animal-specific waveform libraries. Furthermore, RR intervals of all individual beats were analyzed to identify irregularities consistent with ventricular arrhythmias (e.g. premature beats, post-extrasystolic pause). Ventricular arrhythmic events were classified according to the guidelines of The Lambeth Conventions^29–31^ by an operator blinded to the experimental groups. Controversial ECG segments were classified by 3 independent ECG experts.

Premature ventricular beats (PVB) were identified by the presence of at least 2 of the following 3 criteria:

1. atypical QRS configuration with alteration of the T-wave
2. atrioventricular dissociation
3. compensatory post-extrasystolic pause

2 or 3 consecutive PVBs were defined as couplets or triplets, a run of 4 or more consecutive PVBs were defined as ventricular tachycardia (VT). Ventricular fibrillation (VF) was identified by the absence of distinguishable individual QRS complexes.

### Echocardiography

Transthoracic echocardiography was performed using the Vevo 2100 system (VisualSonics, Toronto, Canada). Animals were anesthetized with isoflurane (1.5–2%) and positioned on a pre-heated panel. 2D short and long axis views were recorded at the mid-papillary and the aortic valve level, respectively. Measurements were received from B-mode recordings. Ejection fraction (EF) and diastolic left ventricular diameter (LVIDd) were recorded according to standard procedures. Analysis was performed using the Vevo 2100 software by an operator blinded to the experimental group.

### Data management and statistics

Data was analyzed using GraphPad Prism (version 6.0, Graphpad, La Jolla, CA, USA). Data is given either as median and 5–95 percentile, as median with interquartile range or as mean ± SEM, as stated in the text. Samples were tested for normal distribution. Parametric or nonparametric tests were calculated and corrected for multiple comparisons using one-way ANOVA followed by a Newman-Keuls test, two-way ANOVA followed by a Sidak test, or Kruskal-Wallis test followed by a Dunn’s test, with an α-level at 0.05. The datasets generated during and/or analyzed during the current study are available from the corresponding author on reasonable request.
